# Effect of non-human hosts on the human biting rate of primary and secondary malaria vectors in Tanzania

**DOI:** 10.1186/s12936-023-04778-x

**Published:** 2023-11-08

**Authors:** Godfrey C. Katusi, Marie R. G. Hermy, Samwely M. Makayula, Rickard Ignell, Ladslaus L. Mnyone, Sharon R. Hill, Nicodem J. Govella

**Affiliations:** 1 Department of Environmental Health and Ecological Sciences, Ifakara Health Institute, Off Mlabani Passage, P.O. Box 53, Ifakara, Morogoro Tanzania; 2 Department of Microbiology, Parasitology and Biotechnology, College of Veterinary Medicine and Biomedical Sciences, Sokoine University of Agriculture, P.O. Box 3019, Morogoro, Tanzania; 3 Disease Vector Group, Unit of Chemical Ecology, Department of Plant Protection Biology, Swedish University of Agricultural Sciences, P.O. Box 190, 234 22 Lomma, Sweden; 4 Institute of Pest Management, Sokoine University of Agriculture, P.O. Box 3110, Morogoro, Tanzania; 5 School of Life Sciences and Bioengineering, Nelson Mandela African Institution of Science and Technology, Arusha, Tanzania

**Keywords:** *Anopheles*, *Culex*, Zooprophylaxis, Zoopotentiation, Host preference, Cattle

## Abstract

**Background:**

Malaria vectors vary in feeding preference depending on their innate behaviour, host availability and abundance. Host preference and human biting rate in malaria vectors are key factors in establishing zooprophylaxis and zoopotentiation. This study aimed at assessing the impact of non-human hosts in close proximity to humans on the human biting rate of primary and secondary malaria vectors, with varying host preferences.

**Methods:**

The effect of the presence of non-human hosts in close proximity to the human host on the mean catches per person per night, as a proxy for mosquito biting rate, was measured using mosquito-electrocuting traps (METs), in Sagamaganga, Kilombero Valley, Tanzania. Two experiments were designed: (1) a human versus a calf, each enclosed in a MET, and (2) a human surrounded by three calves versus a human alone, with each human volunteer enclosed individually in a MET spaced 10 m apart. Each experiment was conducted on alternate days and lasted for 36 nights per experiment. During each experiment, the positions of hosts were exchanged daily (except the human in experiment 2). All anopheline mosquitoes caught were assayed for *Plasmodium* sporozoites using enzyme-linked immunosorbent assay.

**Results:**

A total of 20,574 mosquitoes were captured and identified during the study, of which 3608 were anophelines (84.4% primary and 15.6% secondary malaria vectors) and 17,146 were culicines. In experiment 1, the primary malaria vector, *Anopheles arabiensis,* along with *Culex* spp. demonstrated a preference for cattle, while the primary vectors, *Anopheles funestus*, preferred humans. In experiment 2, both primary vectors, *An. arabiensis* and *An. funestus*, as well as the secondary vector *Anopheles rivolurum*, demonstrated behaviours amenable to zooprophylaxis, whereas *Culex* spp. increased their attraction to humans in the presence of nearby cattle. All anopheline mosquitoes tested negative for sporozoites.

**Conclusions:**

The findings of this study provide support for the zooprophylaxis model for malaria vectors present in the Kilombero Valley, and for the zoopotentiation model, as it pertains to the *Culex* spp. in the region. However, the factors regulating zooprophylaxis and zoopotentiation are complex, with different species-dependent mechanisms regulating these behaviours, that need to be considered when designing integrated vector management programmes.

## Background

The host-feeding preference of malaria vectors is complex and may be modulated by the access and availability of preferred hosts, as well as the abundance of alternative hosts [1–4]. For example, using blood meal analysis, the highly anthropophilic *Anopheles gambiae *sensu stricto (*s.s.*) has been demonstrated to feed more frequently on non-human hosts in areas where human hosts are not readily accessible [3, 5–7]. In contrast, *An. gambiae s.s.* and *Anopheles pharoensis* exhibit greater anthropophily during direct side-by-side experimental comparison between human and non-human hosts [3, 4]. The demonstrated variability in host selection by vectors generally considered anthropophilic in the broader literature, may either increase or decrease the risk of transmission through changing human-vector contact, which is referred to as zoopotentiation (increased contact) or zooprophylaxis (decreased contact) [1, 8]. The usefulness, as well as the potential risks, associated with control strategies based on zooprophylaxis is fundamentally linked with the degree of host preference demonstrated by the local vector communities, and requires a standardized, ethically acceptable, direct measure of host preference in primary and secondary vectors at a local and regional level.

Host-feeding patterns determine both the frequency of blood feeding by vectors and the ability of the vector to transmit disease agents [9–11]. Changes in host preference in response to the use of control interventions and changes in host availability have been demonstrated for several primary malaria vectors, including *Anopheles arabiensis*, *Anopheles funestus s.s.* and *An. gambiae s.s.* [12, 13]. While many *An. funestus s.s.* and *An. gambiae s.s.* populations continue to demonstrate their ancestral anthropophagic, endophilic and endophagic behaviours [7, 14–16], these and other species, including *An. arabiensis*, currently the predominant malaria vector in sub-Saharan Africa [12, 17, 18], are reported to increasingly vary their patterns of blood feeding on hosts depending on host availability, particularly in the presence of cattle [2, 8, 19–22]. There is thus a need for regular surveillance of host-feeding preference in not only primary, but also secondary malaria vectors, to assess how these behavioural changes may affect the efficacy of current vector control tools, and the risk of disease transmission. As moving towards the malaria elimination target of 2030 set for Tanzania [23], the host feeding patterns of even the seemingly less important secondary vectors that are critical in sustaining transmission should be addressed.

Malaria vectors in the Kilombero valley, and other parts of Tanzania, are exhibiting alterations in patterns of human feeding similar to that observed in other malaria endemic regions of Africa, regulated by the availability and abundance of alternative hosts, such as cattle [12–14, 24–26]. However, as in most malaria endemic areas, information on the extent of variation in anthropophagy is either mostly lacking, or nor regularly updated [27]. Major reasons for this include the lack of reliable and ethically acceptable tools for the direct assessment of mosquito biting rate, as well as a lack of funding to conduct studies in many localities for a more comprehensive conclusion on vector bionomics. To overcome the first challenge, recent studies have demonstrated that the mosquito-electrocuting trap (MET) is a viable, sensitive tool for directly assessing mosquito attraction to hosts, and a proxy for human biting rate [13, 28–30]. The current study employed the MET to assess the effect of non-human host (cattle) availability on the human attraction of primary and secondary malaria vectors within the Kilombero valley. How the availability of alternate hosts alters the anthropophagy of malaria vectors, and the implications for disease transmission risk and control, are discussed.

## Methods

### Study area

The study was conducted in Sagamaganga village (S 8° 3′ 50.352″ E 36° 47′ 46.254″), which is situated ca. 17 km from Ifakara town within the Kilombero River Valley, south-eastern Tanzania. The valley experiences an average annual rainfall ranging from 1200 to 1800 mm, and annual temperatures ranging between 20 and 32 °C [31, 32]. There are two main seasons: the wet season, between February to June, and the dry season, from July to January. *Anopheles arabiensis* is the predominant malaria vector in the area, followed by *An. funestus s.s.* and *An. gambiae s.s.* [13, 14, 33–35]. In the Kilombero Valley, the most commonly reported secondary vectors are *Anopheles coustani* and *Anopheles squamosus* [34, 35]. Most malaria cases are caused by *Plasmodium falciparum*, with the rate of prevalence decreasing from 14% in 2007–2011 [32, 36] to 0.4% in 2019 (Swai Kyeba, pers. commun*.*). Most of the residents in the area practice subsistence agriculture, particularly rice and maize cultivation, as well as livestock rearing. Cattle are the most common livestock species in the area, followed by sheep, goats, chickens and dogs [34].

### Effect of non-human host availability on human mosquito biting rate

**Fig. 1:**
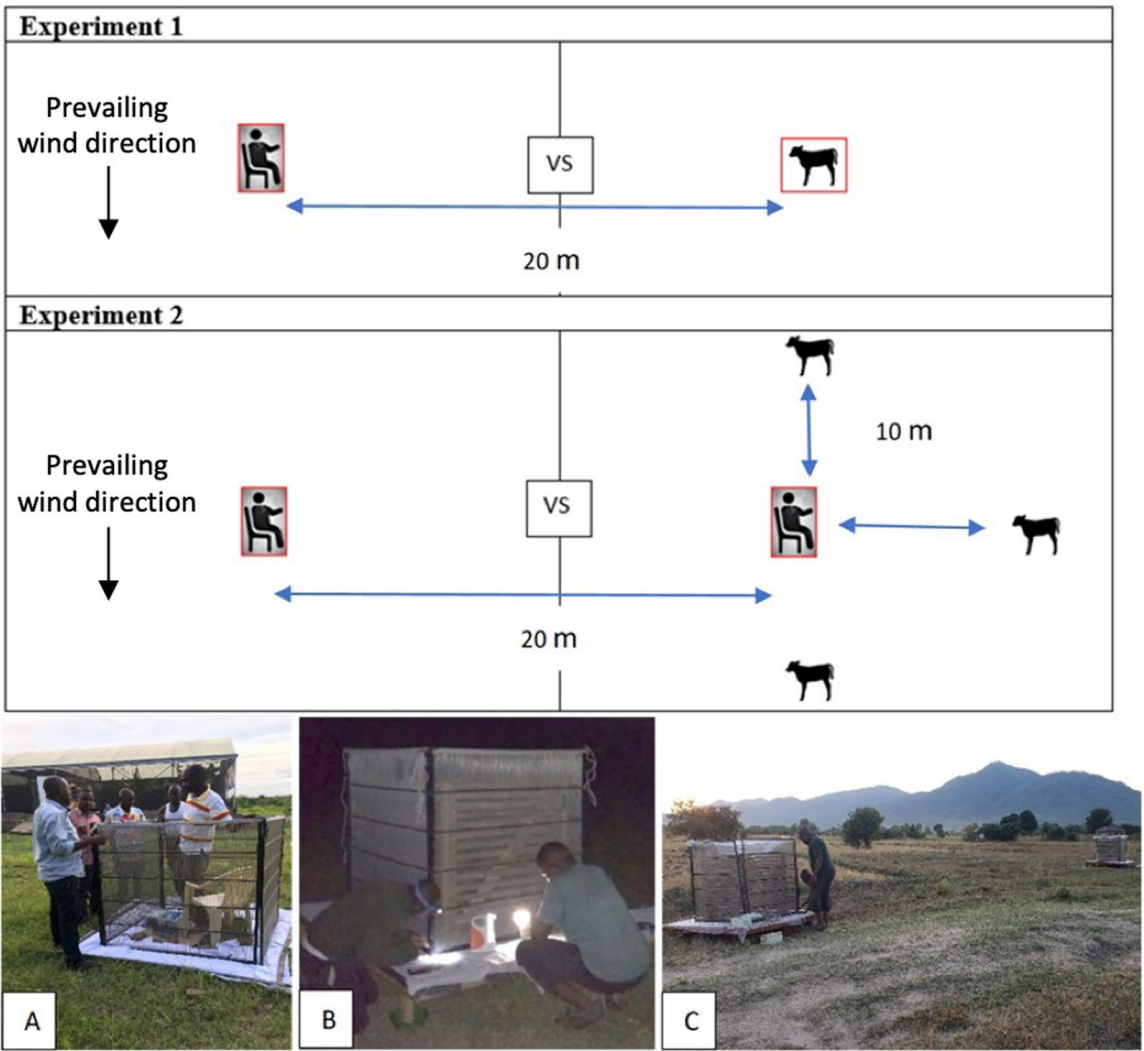
The effect of host availability on human preference. Diagrammatic representation of a two-choice assay with a human volunteer and a calf, each enclosed in mosquito-electrocuting traps (METs; red cubes), set 20 m apart (top panel); and **b** two human volunteers, each enclosed in METs, with one surrounded by three calves, each 10 m from the human volunteer (middle panel). **a** A human volunteer in the MET. **b** The collection of mosquitoes from the white sheets and electric grids around the calf every 45 min, throughout the night. **c** The set-up of the two-choice assay in an open field, 100 m away from human habitation

To assess the mean catches per person per night of primary and secondary malaria vectors, as a proxy for mosquito biting rate, and how this may be modulated by the presence of non-human hosts in near proximity, two experimental designs were used. In the first experiment, a single human and a single calf were each placed in METs (four panels placed in a square, each panel measuring 125 cm width × 122 cm height), which were set 20 m apart in an open field, 100 m away from human habitation, as *per* procedures described by Govella et al*.* and Githu et al*.* [28, 37]. The rationale for the short distance between the METs was to ensure competition between the hosts enclosed within each trap [37]. The traps were deployed so that neither trap was upwind of the other, in relation to the prevailing wind direction (Fig. 1). The treatments were exchanged between the two trap positions daily. Following the determination of host preference for each primary and secondary malaria vector caught in this location in experiment 1, a second experiment was conducted to assess the effect of non-human hosts in near proximity to a human host on the mosquito biting rate. In experiment 2, a single human in a MET was surrounded by three tethered calves, kept at 90° angle to one another and 10 m away from one of the MET-enclosed human volunteers (Fig. 1). A second human volunteer was enclosed in a MET and spaced 20 m apart from the other volunteer (Fig. 1). The rationale for the short distance between the human volunteers, and between the three calves and one of the human volunteers was to ensure interaction among the hosts, without influencing captures in the MET not surrounded by cattle (Fig. 1). The human volunteers were exchanged between the treatments daily. The two experiments were evaluated on alternate nights, for a total of 72 nights, from Dec 2019 to July 2021. For every instance of experiment 1, experiment 2 was conducted on subsequent nights to avoid any seasonal variation in the trap capture rates. The human volunteers weighed on average 70 ± 2 kg, while the calves weighed on average 68 ± 2 kg, in order to control for a similar release rate of host odour. In order to minimize distress, the calves were milk-fed immediately prior to the start of the experiments after returning with their mothers to the overnight enclosure, which delayed the onset of experiments by 1 h, compared to that which is deemed the standard onset time, 18h00, of similar field experiments in this region. Between 19h00 and 06h45, traps were active in 45 min bouts, and then the traps were switched off, the electrocuted mosquitoes collected using forceps and hand-held aspirators, and those which stuck to the surface of the panels were gently removed using a small brush prior to collection. Electrocuted mosquitoes were kept separately, by hour, in labelled paper cups. The voltage for the METs was checked regularly to ensure consistent power throughout the trapping period. Every morning, the collected mosquitoes were transported to the laboratory at Ifakara Health Institute, for morphological and molecular identification.

### Mosquito identification and sporozoite detection

All mosquitoes collected were sorted, counted and morphologically identified in the laboratory with the aid of a dissection microscope [38, 39]. Morphologically, the mosquitoes were identified as members of the *An. gambiae* and *An. funestus* species complexes, as well as *An. coustani*, *Anopheles ziemanni*, *Culex* spp*.* and *Aedes* spp. Moreover, these mosquitoes were classified by sex. All mosquitoes identified as belonging to the *An. gambiae* (n = 2 532) and *An. funestus* (n = 577) species complexes were subsequently identified to species level using multiplexed polymerase chain reaction [40–42]. In addition, circumsporozoite enzyme linked immunosorbent assay (ELISA; IgG identifiers, KPL, Gaithersburg, US) was performed on all primary and secondary malaria vectors (n = 2 828) to detect the presence of malaria parasites as described by Burkot et al*.* and Wirtz et al*.* [43, 44]. To avoid false positives, the ELISA lysates were heated in a water bath at 100 °C for 10 min to inactivate heat-labile antigens other than the *P. falciparum* circumsporozoite proteins [45].

### Statistical analysis

Data handling and analysis was done using R statistical software (version 4.0.2) and JMP® Pro (version 16.0.0, SAS Institute Inc., Cary, NC, 1989–2022). The total number of mosquitoes caught in the two experiments (Tables 1 and 2) were compared by species using nominal regression (JMP® Pro 16.0.0, SAS Institute Inc.). The proportions of primary and secondary malaria vectors captures were compared between traps in experiment 1, volunteer (METh) vs. calf (METc), and experiment 2, volunteer (METh0) vs. calf-surrounded volunteer (METhC), using a chi-squared test (JMP® Pro 16.0.0, SAS Institute Inc.). The mosquito biting rate, reported as mean catches per person per night, were calculated using the *Rmisc* package, and compared for each species using generalized linear mixed models (GLMMs) augmented with the *matrix*, *lattice* and *lme4* packages [46] (R statistical software 4.0.2). Separate analyses was performed for experiment 1 and experiment 2, as well as for each mosquito species collected. Since the data were zero-inflated and over-dispersed (Shapiro test), a negative binomial distribution was employed [46]. The mosquito catches per person per night were treated as a dependent variable, with capturing method fitted as an independent fixed effect, and sampling night and positions of the traps as random effects. To quantify the likelihood associated with each comparison, the relative risks and their respective 95% confidence intervals are reported in Tables 3 and 4. All analyses used a significance level (alpha) of 0.05. Since all mosquitoes from both experiments 1 and 2 were sporozoite negative, no statistical analysis was performed on these datasets.

**Table 1:** The composition of female mosquito species caught by mosquito-electrocuting traps (METs) in experiment 1

Species	METh	METc	Total
*An. arabiensis*^*a*^	708	961	1669
*An. funestus s.s*^*a*^	103	12	115
*An. rivolurum*	9	36	45
*An. leesoni*	6	41	47
*An. funestus s.l*	12	1	13
*An. coustani*	48	132	180
*An. pharoensis*	8	13	21
*Culex* spp.	2593	3687	6280
*Mansonia* spp.	35	38	73
*Aedes* spp.	0	2	2
Total *Anopheles* spp.	894	1196	2090
Total	3522	4923	8445

MET traps baited with a human (METh) vs. MET traps baited with a calf (METc)

^a^Denote primary vectors

**Table 2:** The composition of female mosquito species caught by mosquito-electrocuting traps (METs) in experiment 2

Species	METh0	METhC	Total
*An. arabiensis*^*a*^	499	364	863
*An. funestus s.s*^*a*^	144	99	243
*An. rivolurum*	37	16	53
*An. leesoni*	15	7	22
*An. funestus s.l*	15	24	39
*An. coustani*	33	59	92
*An. pharoensis*	7	17	24
*Culex* spp.	3428	5402	8830
*Mansonia* spp.	2	20	22
*Aedes* spp.	2	0	2
Total *Anopheles* spp.	750	586	1336
Total	4182	6008	10190

MET traps baited with a human (METh0) surrounded by three calves (METhC)

^a^Denote primary vectors

**Table 3:** Comparisons of the daily mean catches per person per night of primary and secondary malaria vectors collected in the METs during experiment 1

	No	Mean catch	RR	Lower	Upper	P-value
*An. arabiensis*						
METh	36	19.67	0.818	0.670	0.999	0.049
METc	36	26.69	1.000	–	–	NA
*An. funestus* s.s						
METh	36	2.86	8.659	4.737	15.828	< 0.001
METc	36	0.33	1.000	–	–	NA
*An. coustani*						
METh	36	1.33	0.364	0.124	1.064	0.065
METc	36	3.67	1.000	–	–	NA
*An. rivolurum*						
METh	36	0.25	0.250	0.058	1.083	0.064
METc	36	1	1.000	–	–	NA
*Culex* spp.						
METh	36	72.03	0.673	0.534	0.849	< 0.001
METc	36	102.42	1.000	–	–	NA

MET traps baited with a human (METh) vs. MET traps baited with a calf (METc). No. denotes the number of replicates. RR denotes relativerelative risk. Upper and Lower denotes the upper and lower confidence intervals, respectively

**Table 4:** Comparisons of the daily mean catches per person per night of primary and secondary malaria vectors collected in the METs during experiment 2

	No	Mean catch	RR	Lower	Upper	P-value
*An. arabiensis*						
METh0	36	13.86	1.000	–	–	NA
METhC	36	10.11	0.744	0.595	0.931	0.010
*An. funestus* s.s						
METh0	36	4	1.000	–	–	NA
METhC	36	2.75	0.684	0.526	0.890	0.005
*An. coustani*						
METh0	36	0.92	1.000	–	–	NA
METhC	36	1.64	1.756	0.554	5.565	0.338
*An. rivolurum*						
METh0	36	1.03	1.000	–	–	NA
METhC	36	0.44	0.426	0.235	0.774	0.005
*Culex* spp.						
METh0	36	95.22	1.000	–	–	NA
METhC	36	150.06	1.590	1.259	2.007	< 0.001

MET traps baited with a human (METh0) surrounded by three calves (METhC). No. denotes the number of replicates. RR denotes relative risk. Upper and Lower denotes the upper and lower confidence intervals, respectively

## Results

### Mosquito catches

**Fig. 2:**
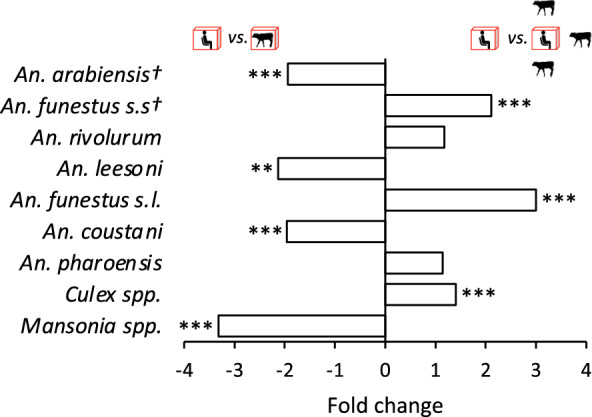
Difference (fold change) in total numbers of mosquitoes caught in experiments 1 and 2. Symbol (†) denotes primary vectors. Asterisks denote the level of significance following a nominal regression analysis (**P < 0.01; ***P < 0.001)

A total of 20754 mosquitoes were captured throughout the study, of which 3610 (3426 females, 184 males) were anophelines and 17146 (15209 females, 1937 males) were culicines. The collected female anophelines consisted of 2890 (84.4%) primary (Tables 1 and 2, asterisks) and 536 (15.6%) secondary malaria vector species (Tables 1 and 2). The presence of cattle surrounding one volunteer significantly affected the total numbers of mosquitoes caught in experiment 2 compared with experiment 1 in a species-specific manner (Fig. 2).

### Experiment 1

To investigate mosquito host preference, the number of mosquitoes for each species caught in human- and calf-baited competing METs were analysed. The proportion of captured primary and secondary malaria vectors varied significantly between METc and METh (χ_1_^2^ = 35.129, P < 0.001) in the two-choice experiment. For the most abundant primary malaria vector in the area, *An. arabiensis*, the mean number of mosquitoes caught in METh was significantly lower than that caught in METc (Table 3). In contrast, the mean number of *An. funestus* caught in METh was significantly higher than that caught in METc (Table 3). While no significant effect of host was found for the secondary malaria vectors, both *An. coustani* and *An. rivolurum* were caught in higher numbers in METc than that in METh (Table 3). Moreover, *Culex* spp. were captured in significantly higher numbers in METc than in METh (Table 3).

### Experiment 2

To evaluate the effect of non-human host presence in close proximity to a human host on mosquito biting rate, the number of mosquitoes for each species caught in human-baited METs, with and without surrounding calves, was analysed. The proportion of captured primary and secondary malaria vectors varied significantly between METh0 and METhC (χ_1_^2^ = 9.9669, P = 0.002), in the dual-choice experiment. For *An. arabiensis*, the mean number of mosquitoes captured in METhC was significantly lower compared to that in METh0 (Table 4). In contrast, *An. funestus*, and *An. rivolurum*, were caught in significantly higher numbers in the METh0 compared to the METhC (Table 4). *Culex* spp. were captured in significantly higher numbers in METhC than in METh0 (Table 4).

## Discussion

By limiting the number of factors associated with livestock-human interactions, the findings presented in this study demonstrate a species-dependent change in biting rate by primary and secondary vectors in the presence of cattle in close proximity. The availability of livestock, which act as dead-end hosts for malaria parasites, has the potential to change the interaction between vectors and humans, thereby modulating malaria transmission. While zooprophylaxis has been advocated as a vital part of integrated vector management, the underlying mechanism driving its effective design is still debated, with the characteristics of the local vectors and the location of the livestock in relation to human dwellings identified as key factors [1, 47]. The findings are discussed in relation to zoopotentiation and zooprophylaxis.

The host preference of mosquitoes exhibited in the two-choice experiment (experiment 1) reflected the anthropophilic response of *An. funestus* [14] and the generally zoophilic/opportunistic behaviour of *An. arabiensis, An. rivulorum* and *Culex* spp*.*, as previously described in Kilombero Valley and beyond [2, 13, 47–49]. In such a scenario, the rationale for implementing zooprophylaxis as a malaria control measure appears valid [1, 8]. By increasing the number of cattle in relation to a single person, the zooprophylaxis model would suggest a reduction in the human biting rate, in favour of mosquitoes feeding on the surrounding cattle [50]. The findings from this study support the zooprophylaxis model for the *An. arabiensis* and *An. rivolurum*, as well as for the anthropophilic *An. funestus s.s*. In all three instances, fewer mosquitoes were caught by the human-baited MET surrounded by calves, compared to a similar MET without cattle positioned in close proximity. While a previous study suggested that the zooprophylactic effect of nearby cattle was dependent on the location of the human host and the malaria vectors present [51], this study demonstrated that this effect was present outdoors for all three species. A comparison between the overall number of mosquitoes caught in experiment 1 and 2, however, suggests that the mechanism underlying the zooprophylactic effect appears to be species dependent. The reduction by half of *An. arabiensis* caught by the human-baited METs in experiment 2 indicates that the calves are likely providing additional hosts for the mosquitoes, and thus reducing the biting rate. The doubling of the number of *An. funestus s.s*. caught during the same experiment, however, correlates with the doubling of its preferred human host, and suggests that the zooprophylaxis is related to the avoidance of cattle odour. The similar overall numbers of zoophilic *An. rivolurum* caught between the two experiments, together with the demonstrated reduction in mosquitoes caught in the human-baited trap surrounded by cattle, indicates that these mosquitoes may be avoiding the combination of human and cattle odour. While data from this study demonstrate that both primary and secondary vectors in the Kilombero Valley are amenable to zooprophylaxis, the mechanism by which this control measure may be effective differs according to vector species, rather than strictly host preference, as suggested by previous studies [1, 4, 12, 13, 52].

Zoopotentiation, an increase in the human biting rate in the presence of potential alternate hosts, appears to be species-dependent and can be a major detractor for the implementation of zooprophylaxis-based vector control strategies in multi-vector environments [4, 53–59]. The findings from this study demonstrated that more of the zoophilic *Culex* spp. [this study, 60–64] were caught in human-baited METs associated with cattle than those without, indicating that using a zooprophylaxis vector control strategy in areas with large numbers of *Culex* spp. will likely increase the human biting rate, and the disease transmission, associated with these species. These results call into question the previous reports that suggest a major role of host preference as a predictor of the potential efficacy of the zooprophylaxis model [1, 52].

All the primary and secondary malaria vectors collected in this study were negative for malaria parasites, supporting the decreased malaria transmission in the region from 14% in the early 2000s to 0.4% in 2019 [33, 37, 65; Swai Kyeba, pers. commun.]. The lack of sporozoite-positive malaria vectors is consistent with reports from this region in recent studies [32]. The decline in malaria prevalence during the last two decades has been suggested to be due to urbanization, improved house construction and the use of LLINs in combination with a livestock-keeping lifestyle in the study area and beyond [32, 65]. The relatively low rate of malaria transmission described suggests that zooprophylaxis may be a useful control strategy in this region toward a further reduction in malaria [62], with the caveat that nuisance biting by *Culex* spp. is likely to remain a problem in the region, and may pose a risk of heightened transmission of diseases these mosquitoes may carry. It should be noted that this study was conducted on a single site thus limiting the generalization of the findings, while providing a clear workflow for the determination of the degree of host preference in local populations in a direct, ethically acceptable manner. While providing a proof-in-principle, this workflow is not cost-effective in the current state, and requires additional modification prior to implementation within established vector control programmes.

## Conclusion

As previous studies indicate, the efficacy of zooprophylaxis as a control method is uncertain in ecosystems with several vectors and human disease agents. This study provides support for the zooprophylaxis model for malaria vectors present in the Kilombero Valley, but also for the zoopotentiation model as it pertains to the *Culex* spp. in the region. The role of host abundance in relation to human biting requires further investigation, particularly into which hosts regulate the observed effects. As a whole, this study emphasizes the complexity of factors regulating the efficacy of zooprophylaxis and highlights the danger of making assumptions concerning its use in controlling multi-vector systems based on previously determined host preference.

## Data Availability

Upon request, the data and materials will be made available under defined conditions expressed in writing through an exchange of letters between parties stipulating those conditions and any agreed limits thereof.

## Abbreviations

WHO: World Health Organization
MET: Mosquito-electrocuting trap
IRS: Indoor residual spraying
LLIN: Long-lasting insecticidal nets
CDC: Centers for Disease Control and Prevention
SSA: Sub-Saharan Africa
